# Comparison Between Preoperative Administration of Methylprednisolone With its Administration Before and During Congenital Heart Surgery on Serum Levels of IL-6 And IL-10

**DOI:** 10.5812/ircmj.8093

**Published:** 2013-02-05

**Authors:** Mohammad Abbasi Tashnizi, Ghasem Soltani, Ali Asghar Moeinipour, Hossein Ayatollahi, Amir Saber Tanha, Lida Jarahi, Alireza Sepehri Shamloo, Nahid Zirak

**Affiliations:** 1 Department of Cardiac Surgery, Imam Reza Hospital, Mashhad University of Medical; 2 Departments of Anesthesiology, Imam Reza Hospital, Mashhad University of Medical Sciences, Mashhad, IR Iran; 3 Department of Anatomical and Clinical Pathology, Imam Reza Hospital, Mashhad University of Medical Sciences, Mashhad, IR Iran; 4 Departments of Community Medicine, Mashad University of Medical Sciences, Mashad, IR Iran

**Keywords:** Cardiopulmonary Bypass, Heart Defects, Congenital, Interleukins, Inflammation

## Abstract

**Background:**

Steroid administration during cardiopulmonary bypass is considered to improve cardiopulmonary function by modulating inflammations caused by bypass.

**Objectives:**

This study was performed to compare effectiveness of preoperative and intraoperative methylprednisolone (MP) to preoperative methylprednisolone alone in post bypass inflammatory (IL-6) and anti-inflammatory (IL-10) factors.

**Patients and Methods:**

Fifty pediatric patients undergoing cardiopulmonary bypass surgery from August 2011 to 2012 in the cardiac surgery department of Imam Reza Hospital, the major center for CPB, in Mashhad, Iran were randomly assigned to receive preoperative and intraoperative MP (30 mg/kg, 4 hours before bypass and in bypass prime, number 25) or preoperative MP only (30 mg/kg, number 25). Before and after bypass, four and 24 hours after bypass, serum IL-6 and IL-10 were measured by ELISA.

**Results:**

In both groups, no significant difference with variation of expression for IL-6 (inflammatory factor) and IL-10 (anti-inflammatory factor) in different times after bypass was observed.

**Conclusions:**

No significant difference in reducing post bypass inflammation between preoperative steroid treatment and combined preoperative and intraoperative steroid administration reported and they had the same effects.

## 1. Background

Treatment of congenital heart disease in children often requires CPB(Cardiopulmonary bypass) and myocardial ischemia can induce post cardiac surgery systemic inflammatory response that can lead to organ dysfunction and morbidity (1, 2) and it also induces a systemic cytokine release (3). Clinical manifestations of this inflammatory response in the postoperative period may include fever, low cardiac output, pulmonary insufficiency, fluid retention, coagulopathy, renal dysfunction and neurological disorders (4-7). This inflammatory response requires various anti-inflammatory strategies such a bypass circuits coated with heparin, ultra-filtration, APO-protein, leukocyte blockers and intraoperative steroids (8-11). Steroids have been used for years to suppress inflammation after surgery. Some of studies showed that the use of glucocorticoids can reduce the interleukins 6 and 8, c3a, tumor necrosis factor–(TNF- α) and increase anti-inflammatory cytokines such as IL-10, IL-4 (12). Recently, Considerable attention is on therapeutic interventions aimed at reducing the inflammatory response during CPB. Many studies have shown the increase of serum levels of IL-6 and TNF-α and decrease of IL-10 during and after CPB (13, 14). But there are few studies of steroid treatment advantages in congenital heart surgeries, however one of this studies showed that children receiving a single dose of dexamethasone 1 hour before bypass had lower troponin, IL-6, and TNF-α levels as results of steroid treatments after surgery (4). In other report, compared with intraoperative steroid treatment alone and combined preoperative and intraoperative steroid administration, attenuates inflammatory mediator expression more effectively and it is associated with improved indexes of o2 delivery in the first 24 hours after congenital heart surgery (2). Data from animal studies suggest that prescribed corticosteroid pre and intraoperative may have more anti-inflammatory effects (12). Most of the available studies on adults' heart surgery are limited to the comparison between multi-dose steroid before and during surgery with placebo. There are rare studies that compared multi-dose steroid (before and during surgery) with mono-dose steroid in adults or children before or during bypass; in which the mono-dose were just administrated during the surgery, but we decided to administrate the mono-dose 4 hours before surgery, because It is noticeable that the peak effect of methylprednisolone occurs 1-4 hours after its administration and the duration of action is 12-24 hours, therefore it seems that given an extra dose of methylprednisolone, 1-4 hours before the operation (in addition to routine intraoperative dose) inhibits the inflammatory reaction more than the routine dose (15-17). This inflammatory response after surgery influences on IL-6 as an inflammatory and IL-10 as an anti-inflammatory factors and will change the level of those. Therefore, because of different methods and different effects of steroid administrations, choosing the better methods can lead to reduce the inflammatory response, improvement operation results and reduce deaths.

## 2. Objectives

The aim of this study is to compare systemic inflammatory response by changing the serum level of interleukin 6 and 10, after CPB in patients receiving multi-dose methyl prednisolone (MuD-MP), before and during surgery with patients receiving mono-dose methyl prednisolone (MoD-MP), before the operation.

## 3. Patients and Methods

This study was done in double-blind clinical trial from August 2011 to August 2012 on 50 children under 5 years, who were undergone elective cardiopulmonary bypass surgery due to congenital heart diseases- in the cardiac surgery department of Imam Reza Hospital, the major center for CPB, in Mashhad, Iran. The trial was performed in accordance with the declaration of Helsinki and approved by ethic committee at Mashhad University of Medical Sciences. Parents of patients who were younger than 5 years candidate in elective cardiac surgery by CBP and were willing to make their children involve in the study, were invited and informed about aims, methods and the anticipated benefits of this research. The inclusion criteria was all the patients who candidate for correction of congenital heart defects under CPB, and patients with upper respiratory infection, diabetes, sepsis, GI bleeding, heart arrest a week before surgery, sensitivity to corticosteroids, chronic corticosteroid recipients were excluded. Based on the result of Valerie et al. study, sample size were calculated 25 for each group. Participants sampling was conducted in two steps, at first children were enrolled based on study objective in easy manner, and in second step participants allocated to intervention or control group in a simple random allocation, in which the first patient was placed in group A (using random manner) and other were placed in each of two groups one after one. Parents and data collector were not informed about group allocating. The participants were allocated in two groups, intervention group who received MuD-MP before and during surgery (25 patients) and control group who received MoD-MP before the surgery (25 patients). The intervention group received 30mg/kg intravenous methylprednisolone 4 hours before operation and the same amount of medication at the beginning of the surgery. The control group only received 30mg/kg methylprednisolone in prime liquid 4 hours before operation. The children were placed under general anesthesia by fentanyl, muscle relaxants, and isoflurane. To support the blood circulation, full-flow bypass was used with a moderate hypothermia. During aorta cross-clamp, cold-blood cardioplegia with additional dosing at 20-30 minute intervals was used; ultra-filtration was used in all cases. After operation, the all patients were ventilated with 10-15 ml/kg tidal volume and the ventilation rate was adjusted to control pco2 at 35 mmHg. Patient’s fluid therapy was monitored by the anesthesiologist and diuretic began the day after the surgery. IL-6 and IL-10 levels were measured according to ELISA method before and immediately after operation, 4 and 24 hours after surgery and their changes in two groups. Children’s demographic characteristics, aortic clamping time, pump time, time of postoperative mechanical ventilation, duration of hospitalization in ICU, dosage of inotropic agents were recorded by one of the nurses. All the surgeries were performed by one surgeon, and all experiments by a science laboratory specialist. Data analysis was done by SPSS11.5 software. Quantitative variables normality was determined in Kolmogorov-Smirnov test. T-test, Chi-Square and Mann-Whitney test were used for comparison. The significance level is considered less than 0.05.

## 4. Results 

This study was done on 50 children under 5 years with congenital heart disease who were undergone the elective heart surgery under CBP into two groups of MuD-MP and MoD-MP strategy. Also all the patients in intervention and control groups completed the study. According to two-group ANOVA test, there was no significant difference between the two groups in terms of age, sex, CPB time and the aorta clamping (Table 1).

**Table 1.** Patient Demographics

Groups	Preoperative Methylprednisolone Only	Combined Preoperative/Intraoperative Methylprednisolone	P value
**Age, y, Mean ± SD**	3.7 ± 2.5	3.2 ± 2.1	NS
**Gender, No.**			NS
Male	11	13	
Female	14	12	
**CPB time, min, Mean ± SD**	30 ± 10.8	22 ± 11.3	NS
**Aortic cross-clamp time min, Mean ± SD**	21.5 ± 13.2	23.6 ± 11.3	NS

IL-6 and IL-10 variables, Mann-Whitney test was used which showed no meaningful differences between changes in serum levels of IL-6 and IL-10 in none of the recorded hours (just before the surgery, 0, 4 and 24 hours after surgery) in two groups (Table 2).

**Table 2.** Serum IL-6 and IL-10 level changes before and after CPB (Mean ± SD)

Serum	Preoperative and Postoperative Differences, Mean ± SD	Preoperative and 4 Hours After CBP ^a^ Differences, Mean ± SD	Preoperative and 24 Hours After CBP Differences, Mean ± SD	P value
**IL-10**				NS
Multi-dose methylprednisolone	2.9 ± 1.3	57.4 ± 16.5	20.2 ± 8.2	
Mono-dose methylprednisolone	3.8 ± 2.4	28.3 ± 10.6	14.9 ± 5.6	
**IL-6**				NS
Multi-dose methylprednisolone	34.1 ± 20.2	97 ± 10.9	14.1 ± 2.4	
Mono-dose ethylprednisolone	58.1 ± 22.8	69 ± 14.8	20.2 ± 4.7	

^a^Abbreviation: CBP, cardiopulmonary bypass

During this study, all patients were discharged from hospital with no unusual complication. In none of the patients, study medications side effects such as postoperative bleeding, hypertension, gastrointestinal bleeding and severe disorders water and electrolytes were not observed.

## 5. Discussion

The results of our study indicated that there are no differences in multi-dose or mono-dose effect of methylprednisolone on reduction of the inflammatory responses after the operation. Inflammatory factor IL-6 and anti-inflammatory factor IL-10 had no meaningful statistical differences in the MuD-MP and MoD-MP strategy. In a study conducted by Dr. Valerie et al. (2) on 29 patients in 2003, multi-dose (before and during surgery) and mono-dose (during surgery) were compared. The results showed that prescription of multiple doses was more effective compared with mono-dose on decreasing IL-6 and increasing IL-10 levels. It should be noted that in our study, mono-dose was applied before surgery, while in the study of Valerie it was used during the surgery which can be a reason for the differences in reported results. In Valerie et al. study, there was no differences between the two groups in IL-6 level before surgery (or before receiving corticosteroids), but our study showed differences between the two groups regarding IL-6 levels. Thus, in order to investigate the changes of IL-6 in the hours after the surgery, the difference between the IL-6 level after and before surgery (before steroids) was used in each group. In the Valerie et al. studies, there was a significant difference between the two groups in terms of serum IL-6 and IL-10 immediately and 4 hour after surgery which indicated a greater impact of corticosteroids in multi-dose group than mono-dose group, respectively. In another study by Bronicki et al. (4) the level of IL-6 was investigated on 15 patients who have received 1mg/kg dexamethasone compared with the control group which have received saline one hour before CPB. The level of IL-6 was reduced significantly compared to control group 10 minutes after stopping CPB. In our study, the level of IL-6 showed an increase 4 hours after surgery compared to before surgery in both multi-dose and single-dose groups, but there was no statistical differences between the groups; also IL-6 level changes had not significant differences 24 hours after surgery in both multi-dose and mono dose groups compared to before surgery. In Valerie et al. study, evidences also suggest a return of IL-6 observed level to its level before surgery and there were no significant differences between the two groups. The limitations of this study can be referred to the low sample size and not conducting research on a specific cardiac defect. Therefore, it is recommended that the results should be approved by large multi-centric studies in a specific type of cardiac defect. In this study, the results have not been studied in a special clinical surgery which needs to do in another study by our research group. Also, it is recommended that in order to investigate the effects of steroids prescription, it's better to use other factors such as complements, TNF, quinine peptides and platelet-activating factors as well. Since changes of IL-10 and IL-6 levels showed no significant statistical differences in multi-dose and mono dose methylprednisolone groups at different hours compared to before surgery, so , generally there was no difference in terms of the effects of multi-dose corticosteroids compared with mono-dose corticosteroids on the inflammatory responses and the observed results in our study.
